# TFF-Net: A Feature Fusion Graph Neural Network-Based Vehicle Type Recognition Approach for Low-Light Conditions

**DOI:** 10.3390/s25123613

**Published:** 2025-06-09

**Authors:** Huizhi Xu, Wenting Tan, Yamei Li, Yue Tian

**Affiliations:** School of Civil Engineering and Transportation, Northeast Forestry University, Harbin 150040, China; twt@nefu.edu.cn (W.T.); liyamei0311@126.com (Y.L.); ty@nefu.edu.cn (Y.T.)

**Keywords:** vehicle type recognition, low-light condition, graph attention network, YOLO

## Abstract

Accurate vehicle type recognition in low-light environments remains a critical challenge for intelligent transportation systems (ITSs). To address the performance degradation caused by insufficient lighting, complex backgrounds, and light interference, this paper proposes a Twin-Stream Feature Fusion Graph Neural Network (TFF-Net) model. The model employs multi-scale convolutional operations combined with an Efficient Channel Attention (ECA) module to extract discriminative local features, while independent convolutional layers capture hierarchical global representations. These features are mapped as nodes to construct fully connected graph structures. Hybrid graph neural networks (GNNs) process the graph structures and model spatial dependencies and semantic associations. TFF-Net enhances the representation of features by fusing local details and global context information from the output of GNNs. To further improve its robustness, we propose an Adaptive Weighted Fusion-Bagging (AWF-Bagging) algorithm, which dynamically assigns weights to base classifiers based on their F1 scores. TFF-Net also includes dynamic feature weighting and label smoothing techniques for solving the category imbalance problem. Finally, the proposed TFF-Net is integrated into YOLOv11n (a lightweight real-time object detector) with an improved adaptive loss function. For experimental validation in low-light scenarios, we constructed the low-light vehicle dataset VDD-Light based on the public dataset UA-DETRAC. Experimental results demonstrate that our model achieves 2.6% and 2.2% improvements in mAP50 and mAP50-95 metrics over the baseline model. Compared to mainstream models and methods, the proposed model shows excellent performance and practical deployment potential.

## 1. Introduction

With the accelerated development of socio-economic conditions and the continuous improvement in living standards, the ownership of private vehicles has increased exponentially [1]. The proliferation of private vehicles has created challenges for urban transportation, and the ITS has become a key response [2]. Vehicle recognition technology is the foundation of the ITS, but it still faces obstacles in low-light conditions. Most accidents (68.8%) occur at night [3], and the relatively high risk and percentage of accidents at night highlights the importance of traffic management and safety in low-light environments [4], where accurate and efficient low-light vehicle recognition techniques are urgently needed.

Camera sensor-based vehicle type recognition is a crucial yet challenging task [5], particularly under low-light conditions. Insufficient or uneven illumination reduces image quality [6], obscuring critical visual information such as vehicle contours and texture features [7]. In such scenarios, traditional feature extraction methods struggle to capture vehicle characteristics accurately. Additionally, the low contrast between vehicles and complex urban backgrounds in nighttime environments exacerbates recognition difficulties. Intense light from nearby vehicle headlights and reflections from vehicle surfaces often mask critical morphological features, creating misleading visual artifacts. The light interference can have a negative impact on the accuracy of the recognition.

As vehicle appearances diversify and real-world scenarios grow increasingly complex, traditional methods face limitations in accuracy, real-time performance, and robustness due to their reliance on simplistic image features [8] and the inability to efficiently process large-scale data. Convolutional neural networks (CNNs) are a key technique for extracting image features in deep learning. They can automatically extract multilevel features from image data with a strong generalizability [9]. CNNs primarily focus on a localized feature extraction through stacked network layers, often neglecting global spatial dependencies and semantic correlations. In contrast, GNNs specialize in capturing global relational patterns [10], and common GNN models include graph convolutional neural networks (GCNs) [11], graph attention networks (GATs) [12], and Graph Spatio-Temporal Networks [13]. Current research indicates that GNNs remain underexplored for vehicle type recognition under low-illumination conditions.

To address these challenges, we propose a GNN-based method for vehicle recognition under low-light conditions. GNNs demonstrate a superior capability in capturing semantic relationships and contextual information compared to traditional convolutional architectures. Vehicle images are represented as graph-structured data, where vehicle components and environmental elements constitute interconnected nodes. The proposed method can aggregate local features and global contextual information more efficiently, which is crucial for improving the accuracy of vehicle models’ recognition in under-illuminated environments. The primary contributions of this paper are to

1. Propose a local–global dual-stream architecture with complementary feature learning mechanisms. The local branch introduces a multi-scale convolution kernel with jump connections and ECA-enhanced channel attention, while the global branch employs a novel nine-layer independent convolution design. The dual-stream design effectively integrates local details and global semantics to enhance feature characterization under complex lighting.
2. Design a hybrid GCN-GAT architecture with spatial semantic fusion. Construct a spatial neighbor graph based on dual-stream features and mine spatial and semantic associations among nodes. Combine the attention pooling module for the weighted fusion of global and local features, which significantly improves the discriminative properties of multi-scale features in low-illumination scenes.
3. Develop an adaptive AWF-Bagging algorithm with a dynamic weighting mechanism to enhance the stability of the model through the dynamic allocation mechanism of the basic classifier’s performance. The design of dynamic cross-entropy loss with class balance regularization, combined with the label smoothing technique, reduces the class imbalance bias.
4. Integrate the proposed model into the YOLOv11n detection framework. Classification head reconstruction and the loss function design improve the model’s mAP on the nighttime dataset to 91.4%, with only 4.26 M parameters required, which balances a high accuracy with the feasibility of deployment in edge computing devices.

## 2. Related Works

### 2.1. Vehicle Type Recognition Methods

Vehicle type recognition is a task based on a vehicle’s external features. It relies on identifying and analyzing a vehicle’s external features to assign it to a specific category. Approaches to vehicle type recognition are usually categorized into traditional methods and deep learning-based methods.

#### 2.1.1. Traditional Vehicle Type Recognition Methods

Early research on vehicle type recognition relied heavily on manual means to extract vehicle features and combined feature extraction techniques with various classifiers. Fu H et al. [14] proposed a robust vehicle classification method based on a hierarchical multi-SVM and voting correction scheme to solve vehicle recognition challenges in congested traffic scenarios. A car manufacturer recognition framework combining inter-frame differencing, a symmetric filter, and SIFT algorithm was proposed in the literature [15], which can effectively detect the frontal view of a moving car and realize high-precision recognition. Chaudhary et al. [16] innovatively combined photonic radar technology with support vector machine classification to enhance multi-target detection in complex traffic scenarios. The literature [17] proposes a new supervised dimensionality reduction method, LDA-DE, which reduces the dimensionality by computing the class label dispersion matrix and the diagonal eigenvalue matrix, and it includes techniques for de-emphasis, anti-feature coverage, and outlier removal. With complex and variable weather conditions, Zhang et al. [18] performed a super-pixel segmentation operation in the potential vehicle region, fused enhanced HOG features, and used SVM classification after eliminating overlapping areas. Conventional approaches have a low recognition accuracy in complex traffic scenarios and a limited generalizability.

#### 2.1.2. Deep Learning-Based Vehicle Recognition Methods

Continuous advances in deep learning have fueled the boom in CNN-based techniques, allowing them to gradually take center stage. CNNs avoid the tedious step of manually designing complex feature extractors and show significant performance advantages in vehicle recognition tasks. Alghamdi et al. [19] proposed a hybrid model combining a pre-trained VGG16 convolutional neural network with genetic algorithm feature selection, which in turn leads to the accurate recognition of vehicle classes. The literature [20] achieved high-accuracy vehicle recognition in complex environments through the steps of contrast enhancement, Mask-R-CNN feature segmentation, feature extraction, and autoencoder feature selection. An enhancement of the recognition performance by enhancing the information feature map and reducing the network entropy was also presented in the literature [21]. Dong X et al. [22] extracted local features and introduced a sparse attention module through CNN-In D branching to improve the detailed feature representation of vehicle recognition methods. To enhance the robustness of the vehicle type recognition algorithm, the literature [23,24] proposes an enhanced YOLO detection algorithm to improve the model’s ability to sense vehicle targets. Deep learning has achieved significant results in vehicle recognition. However, CNN-based image recognition methods are constrained by data labeling. The recognition accuracy of CNN models decreases dramatically in the face of different lighting conditions, vehicle occlusion, and deformation.

### 2.2. Graph Neural Network-Based Image Recognition Methods

A large amount of non-Euclidean spatial data exists in the real world, including traffic and transportation networks, social networks, etc., collectively known as graph-structured data [25]. Graph data contains unordered nodes, which makes it difficult to efficiently extract graph structure features with traditional CNNs and pooling. The GNN [26] was first introduced in 2005 to address the challenges traditional neural networks face when dealing with graph data. GNNs have been gradually introduced into pattern recognition [27], text categorization [28], traffic prediction [29], etc., and they exhibit excellent adaptability.

The wide range of applications of GNNs in graph data processing reveals their great potential. Ganesan et al. [30] developed an innovative Fine-Grained Feature Descriptor (FGFD) module to improve the efficiency of image classification at a finer granularity level with the help of GNNs. A label-aware bi-graph neural network was proposed in the literature [31]. The network achieves efficient multi-label fundus image classification by constructing population and pathology-based graph representations. Hanachi et al. [32] learned potential representations through the MVGAE fusion of multi-view graphs to improve face recognition classification performance using labeled and unlabeled data. An homology-oriented bi-kernel graph neural network BKGNN was developed for efficient Human–Computer Interaction classification by Hu et al. [33].

### 2.3. Hybrid CNN-GNN Methods

The simultaneous employment of CNNs and GNNs captures the dependencies between features [34], enhances features’ representation and achieves good results in tasks such as image recognition [35] and change detection [36]. A hybrid multimodal CNN-GNN framework for single-step moving traffic prediction has been proposed in the literature [37]. The framework used CNNs constructed via ConvLSTM and AGCN outputs using adaptive graph convolutional networks. The framework achieved a prediction error of less than 10 baselines based on a real dataset. MLRSSC-CNN-GNN [38] utilized CNNs to learn high-level appearance features in a scene and constructed them as a scene graph. A multi-layer integrated GAT was designed to solve the problem of multi-label remote sensing image scene-classification. Cui et al. [39] used GCNs and CNNs to learn large-scale irregular region and small-scale regular region map features, respectively. A graph-based dual convolutional network generates complementary spatio-temporal spectral features for automatic road extraction from high-resolution remote sensing images. Brain-GCN-Net [40] achieved a more comprehensive representation of brain tumor images by integrating CNN and GNN architectures. The results demonstrated that the proposed model outperforms existing pre-trained models and traditional CNN architectures.

Hybrid CNN-GNN methods achieve better results compared to single methods and show great potential. CNNs and GNNs have their own unique advantages in processing image data. With the vehicle image transformed into a graph structure, the GNN can accurately capture the contextual information of the vehicle and its surroundings to extract more discriminative features. The research into hybrid CNN- and GNN-based methods can compensate for the shortcomings of existing vehicle type recognition methods in low-light environments. The proposed method provides strong support for developing intelligent transportation systems and improving road safety.

## 3. Method

**Figure 1:**
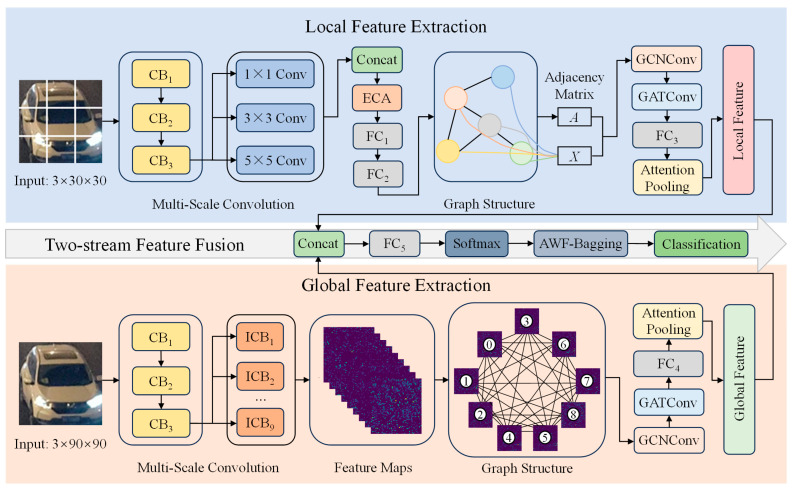
General structure of the TFF-Net model.

This paper proposes a dual-stream low-light vehicle type recognition model based on GNNs named TFF-Net. The overall model structure is shown in Figure 1. TFF-Net fuses multi-scale CNN features with hybrid GNN topology-aware graph information to improve recognition performance in complex scenes by collaboratively modeling local and global features.

The local branch (Net1) employs multi-scale convolutional kernels and a channel attention mechanism for fine-grained feature extraction from the segmented image of the nine-box grid. The global branch (Net2) captures contextual information such as contour and color distribution through independent convolutional layers and maps features to fully connected graph nodes. The GNN module combines a GCN and GAT to mine spatial and semantic associations between nodes to generate abstract high-level graph features. The attentional pooling module performs a weighted fusion of global and local features, suppresses the interference of irrelevant information, and finally outputs the classification results through the fully connected layer. For the category imbalance problem, the model introduces dynamic weight adjustment and label smoothing techniques to optimize the loss function. The AWF-Bagging algorithm integrates multiple base classifiers to further improve the robustness and accuracy.

### 3.1. Two-Stream Feature Extraction

#### 3.1.1. Local Feature Extraction

**Figure 2:**
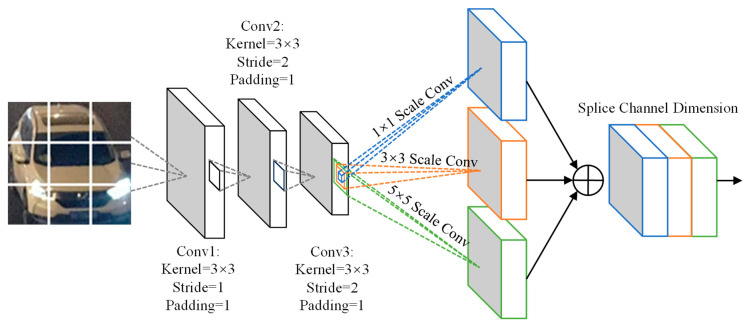
Local feature extraction network, Net1.

This paper uses a region segmentation method to uniformly divide the input image into 3 × 3 grid regions. This method can force the model to focus on key local structures such as headlights and air intake grilles, thus capturing detailed vehicle local features based on spatial structure. Based on this design, the multi-scale local feature extraction network Net1 uses a three-layer convolutional architecture. As shown in Figure 2, each convolutional layer is followed by a batch normalization, ReLU activation function, and Dropout layer to prevent overfitting. To enhance its feature discrimination ability in complex backgrounds, the model introduces a multi-scale feature fusion module to handle the output of the third-layer convolution. The module deploys three different scales of convolutional kernels, 1 × 1, 3 × 3, and 5 × 5, in parallel to realize a reduction in channel dimensionality, local texture extraction, and perception of large-scale structures, respectively. Subsequently, the multi-scale output is stitched along the channel dimensions to form a fused feature map.

**Figure 3:**
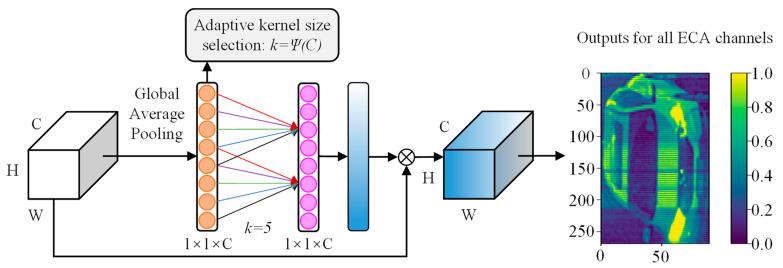
ECA channel attention mechanism.

To further enhance the feature representation, the network embeds the ECA channel attention module [41] after the fusion layer, the structure of which is shown in Figure 3. The module dynamically calibrates the feature weights of each channel through a lightweight attention mechanism to enhance the feature representation of key channels. After the above processing, the hierarchical feature vectors of the nine grid regions are output. These local features with spatial location information will provide rich node feature representations for the subsequent construction of the graph neural network.

#### 3.1.2. Global Feature Extraction

**Figure 4:**
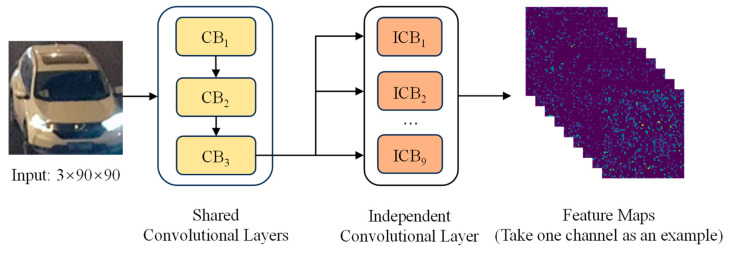
Global feature extraction network, Net2.

Due to the influence of complex lighting conditions and a variable vehicle morphology in low-light environments, the traditional single-feature extraction method finds it challenging to satisfy practical needs. Based on the feature map *X*_3_ output from the shared convolutional layer, this paper designs the global feature extraction network Net2, illustrated in Figure 4. The network utilizes a parallel architecture of nine independent convolutional layers. Each convolutional layer has a unique parameter configuration that enables a multifaceted parsing of the input feature maps from different viewpoints. The independent convolutional layer performs feature extraction on the feature map $X_{3}$ through differentiated convolutional kernels to capture diverse global feature information such as the overall vehicle contour, color distribution, and spatial layout.

The output of the jth independent convolutional layer is calculated in Equation (1).

(1) $$X_{gj}=\mathrm{Dropout}(\mathrm{ReLU}(\mathrm{BatchNorm}(W_{gj}*X_{3}+b_{gj})))$$

where ∗ denotes the convolution operation; $W_{gj}$ denotes the convolution kernel weight; and $b_{gj}$ denotes the convolution kernel bias.

Each independent convolutional layer performs a sliding convolution operation on top of the feature map through different convolutional kernels to extract diverse features. During the training process, the convolutional kernel parameters are dynamically adjusted based on backpropagation so that each convolutional layer can adaptively learn feature representations at different scales.

### 3.2. Feature Fusion Based on Graph Structure Relationship Modeling

#### 3.2.1. Graph Structure Construction

**Figure 5:**
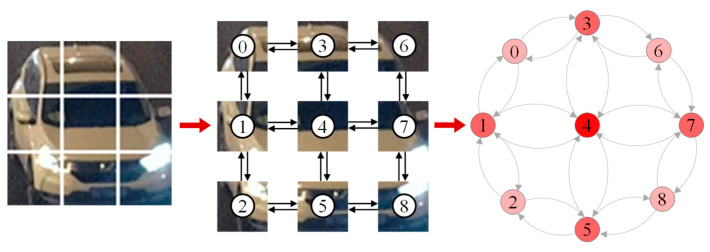
Local graph structure.

The construction of fully connected graph structures based on spatial adjacencies allows for the modeling and analysis of image topology using graph neural networks. As shown in Figure 5, the segmented 3 × 3 grid is used as nodes, labeled with ordinal numbers, and edge connections are established based on spatial adjacencies. The color of the ordinal number indicates the size of the degree. The nine grid image features extracted by Net1 are used as spatial association nodes to construct the local graph structure.

**Figure 6:**
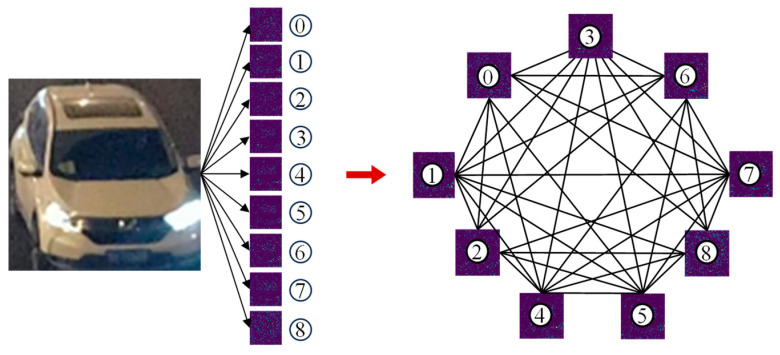
Global graph structure.

The nine feature maps output from Net2 are converted into node feature vectors by flattening them to form a node feature matrix, and the global graph structure indicated in Figure 6 is constructed accordingly. To adequately capture the inter-feature correlation, the adjacency matrix adopts the fully connected form so that information can interact between any nodes.

#### 3.2.2. Graph Neural Network Design

**Figure 7:**
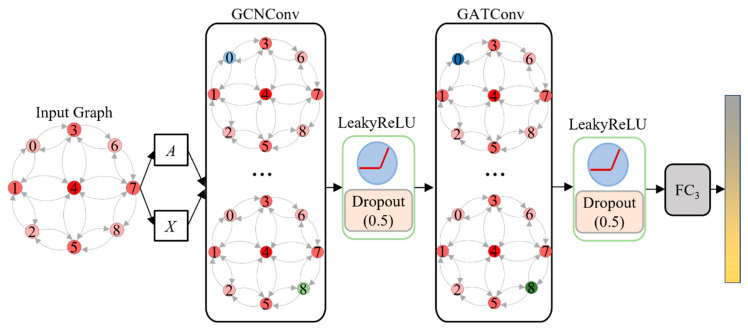
Graph neural network hybrid architecture.

A GCN-GAT hybrid architecture is designed to mine spatial and semantic associations between nodes, dynamically update node features, and entirely mine and integrate advanced features. The GNN structure design is presented in Figure 7. This architecture uses a low computational complexity GCN layer to aggregate neighbor node feature information for the base propagation and initial fusion of features. The GCN does not consider the heterogeneous feature differences between nodes, so a GAT layer capable of an adaptively weighted fusion of neighboring nodes’ features is introduced. With the hybrid use of GCN and GAT layers, the model can learn more abstract and high-level graph features to improve the accuracy and robustness of vehicle type recognition under low-light conditions.

#### 3.2.3. Feature-Weighted Fusion

Subsequently, the attention pooling module weighted and fused the global and local features to suppress the interference of irrelevant information and obtain a more discriminative feature representation.

Normalization of the attention coefficients $\alpha_{g}$ and $\alpha_{l}$ by the Softmax function ensures that the weights satisfy ∑α=1, as follows:

(2) $$\alpha_{g}=\frac{\exp(W_{g}\cdot H_{g})}{\sum \exp(W_{g}\cdot H_{g})}$$

(3) $$\alpha_{l}=\frac{\exp(W_{l}\cdot H_{l})}{\sum \exp(W_{l}\cdot H_{l})}$$

where $W_{g}$ and $W_{l}$ are learnable parameter matrices.

The global feature vector $H_{g}$ and local feature vector $H_{l}$ output from the graph neural network are passed through the attention pooling module to obtain features ${\tilde{H}}_{g}$ and ${\tilde{H}}_{l}$:

(4) $${\tilde{H}}_{g}=W_{g}^{\prime }\cdot H_{g}$$

(5) $${\tilde{H}}_{l}=W_{l}^{\prime }\cdot H_{l}$$

where $W_{g}^{\prime }$ and $W_{l}^{\prime }$ are learnable projection matrices.

Before splicing, global feature $H_{g}$ (of dimension $d_{g}$) and local feature $H_{l}$ (of dimension $d_{l}$) are mapped to the uniform dimension d through the fully connected layer:

After attentional pooling, the aligned features are directly spliced along the channel dimensions without dimensional conflicts as the final fused features vector Z:

(6) $$Z=\mathrm{Concat}(\left[{\tilde{H}}_{g};{\tilde{H}}_{l}\right])$$

The fusion of global and local features after GNN processing can fully utilize the advantages of these two features and reveal their intrinsic connections. Feature fusion provides a more discriminative feature representation for the low-light vehicle type recognition task, thereby improving the model’s effectiveness.

### 3.3. Loss-Regularization Strategy

A more severe dataset category imbalance exists in vehicle type recognition in low-light environments. The sample size of the minority categories is much smaller than that of the majority category. This can lead to model training guided by the traditional cross-entropy loss function, tending to be biased towards the majority category and affecting the generalizability and usefulness of the overall model. In addition, the model is prone to overfitting when learning real labels represented by the one-hot encoding technique, and this makes the model under-adaptive in the face of unknown data. To solve the above problems, we propose an innovative loss function improvement strategy. This strategy introduces the mechanism of the dynamic adjustment of weights based on the original cross-entropy loss function and combines it with the label smoothing technique.

#### 3.3.1. Dynamic Adjustment of Weighting Mechanism

A dynamic adjustment of the weights mechanism is designed, considering the negative impact of category imbalance on model training. The dynamic weights $\omega_{c}(t)$ are calculated as follows:

(7) $$\omega_{c}(t)=\frac{1}{N_{c}(t)}$$

where $N_{c}(t)$ denotes the number of samples in the cth category in the current round t.

During the training process, the categories with small samples correspond to larger weights. The model is forced to pay more attention to these few categories of samples to avoid neglecting a few categories due to category imbalance. Based on the above dynamic weights, the improved loss function is shown in Equation (8).

(8) $$L_{DCE}=-\sum_{c=1}^{C}\omega_{c}(t)\cdot y_{c}\log({\hat{y}}_{c})$$

where C is the number of categories, $y_{c}$ is the one-hot encoding of the true label y of the sample, and ${\hat{y}}_{c}$ is the probability value predicted by the model.

#### 3.3.2. Label Smoothing Technique

In practice, data is often noisy and from anomalous samples. The overconfidence of the model in the training data leads to a degradation in its performance on the test set, hence the introduction of label smoothing techniques [42]. The original true labeling $y_{c}$ is adjusted to smooth the distribution $y_{c}^{\mathrm{smooth}}$:

(9) $$y_{c}^{\mathrm{smooth}}=(1-\varepsilon )\cdot y_{c}+\frac{\varepsilon }{C}$$

where ε denotes the smoothing parameter, which usually takes a value between 0.1 and 0.2, with the actual value of 0.1.

Via smoothing, the true labels are no longer certain but spread to other categories to some extent. This makes the model less dependent on single-category determinism and more robust in its learning process. After incorporating label smoothing in the loss function, a new loss function, $L_{DCE}$, is obtained.

(10) $$L_{DCE}=-\sum_{c=1}^{C}y_{c}^{\mathrm{smooth}}\log({\hat{y}}_{c})$$

The final improved loss function $L_{DLS}$ in Equation (11) is obtained by combining the dynamic weighting mechanism with the label smoothing technique.

(11) $$L_{DLS}=-\sum_{c=1}^{C}\frac{1}{N_{c}(t)}\cdot \left[(1-\varepsilon )\cdot y_{c}+\frac{\varepsilon }{C}\right]\cdot \log({\hat{y}}_{c})$$

After the fused features are fed into the fully connected layer, the model training is optimized by combining the improved cross-entropy loss function to solve the category imbalance problem. This function effectively solves the limitation of traditional cross-entropy loss and provides a more reliable basis for model training and practical applications under complex data distributions.

### 3.4. AWF-Bagging Algorithm Integration

**Figure 8:**
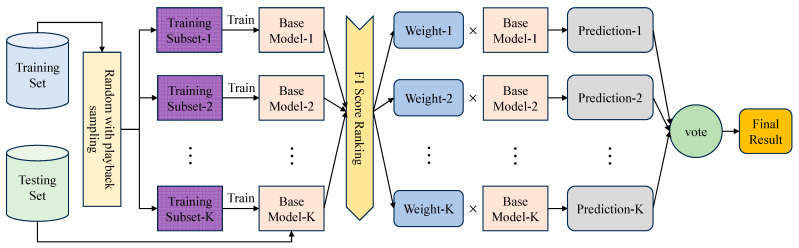
AWF-Bagging integration methodology.

To strengthen the model’s ability to adapt to data heterogeneity and noise, the Adaptive Weighted Fusion-Bagging (AWF-Bagging) algorithm is proposed for multi-classification tasks. Unlike traditional Bagging, where equal weights are averaged or voted, AWF-Bagging assigns differentiated weights to each classifier based on the performance of the base classifiers (F1 scores). Its structure is illustrated in Figure 8.

Obtain the F1 scores of the base classifiers on the test set, rank the F1 scores, and assign the corresponding weights. The weight assignment follows the normalization constraint (sum to 1), which is calculated in Equation (12).

(12) $$\omega_{i}=\frac{F1_{i}}{\sum_{j=1}^{K}F1_{j}}$$

where $\omega_{i}$ means the weight of the ith base classifier, $F1_{i}$ means the F1 score of the ith base classifier, and K means the total number of base classifiers.

High-performance base classifiers are given a higher weight in the integration decision, and low-performance classifiers are suppressed. This mechanism allows the model to automatically adapt to data distribution heterogeneity and differences in the performance of base classifiers rather than relying on fixed rules. Traditional Bagging’s equal-weighted averaging does not allow for this optimization.

For the integration, the predictions of the base classifiers are multiplied by their weights and then accumulated. The final output of the Bagging Heterogeneous Integration Model is obtained as shown in Equation (13).

(13) $$\hat{y}=\sum_{i=1}^{K}\omega_{i}\cdot f_{i}(x)$$

where $\hat{y}$ is the integrated model’s final output, which indicates each category’s probability distribution in the multiclassification task, and $f_{i}(x)$ indicates the prediction result of the ith base classifier on the input x.

A probability threshold, τ, is set to determine whether the input x belongs to a particular category, The specific determination of the category label $C_{L}$ is shown in Equation (14).

(14) $$C_{L}=\left\{\begin{array}{cc} 1,\; & \begin{array}{cc} \mathrm{if} & {\hat{y}}_{c}\geq \tau \end{array}\\ 0,\; & \mathrm{otherwise} \end{array}\right.$$

where ${\hat{y}}_{c}$ denotes the prediction probability of the integrated model for the first category.

AWF-Bagging is based on K base classifiers and uses an adaptive weighted fusion strategy to integrate the prediction results of each base classifier. The constructed feature fusion model is used as a base classifier. K=5 independent base classifiers are constructed. The base classifiers are trained using different training subsets. The base classifiers are trained independently and optimized with hyperparameters to finally generate their respective classifiers $f_{i}(x)$, where i=1,2,⋅⋅⋅,K. In the testing phase, weights $\omega_{i}$ are assigned based on the F1 scores of the base classifiers on the test set. The predictions $f_{i}(x)$ of the base classifiers are weighted and fused to obtain the final output $\hat{y}$ of the integrated model. By setting a probability threshold of τ=0.5, the category to which the input x belongs is determined.

The application of the AWF-Bagging algorithm to the model can effectively utilize the performance difference of the base classifiers to improve the classification accuracy and the model’s robustness.

## 4. Dataset

Vehicle datasets play an essential role in autonomous driving and intelligent transportation. They can provide rich data support for tasks such as vehicle detection, tracking, and classification. Table 1 compares four common publicly available vehicle datasets.

**Figure 9:**
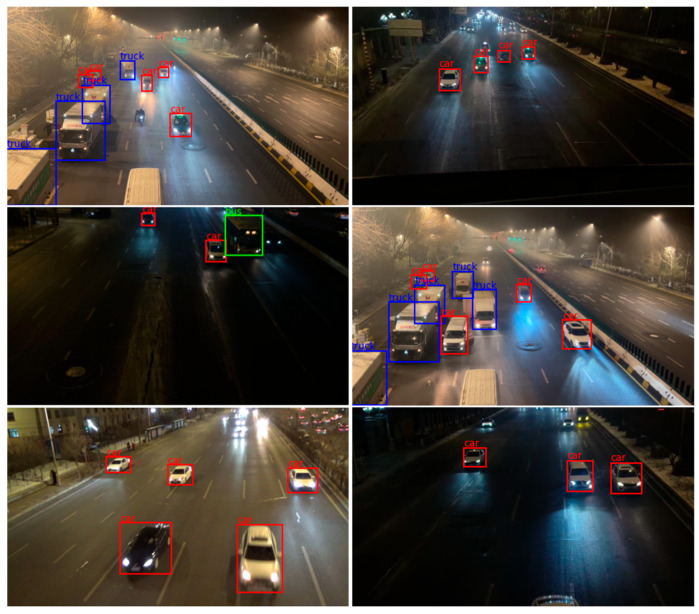
Example of VDD-Light dataset.

The existing datasets have significant shortcomings regarding surveillance viewpoints and the coverage of extreme low-light environments, which hardly reflect complex traffic realities. This study constructs a novel low-light surveillance viewpoint vehicle dataset, VDD-Light. Figure 9 presents the nine images in the VDD-Light dataset and the corresponding labeling results.

A Sony HDR-CX680 camera (Shanghai Suoguang Video Co., Ltd., Shanghai, China.) is used to obtain 26 low-light traffic flow videos by shooting from overhead at the flyover locations of Hexing Road and Xuefu Road sections in Harbin City. The video is disassembled at 30 frames per second, and 5468 standardized images of 3840 × 2160 pixels are filtered. Based on the actual application requirements, we have established a labeling system that includes three types of targets: car, bus, and truck. Image annotation is performed in VOC format and convert to YOLO algorithm-adapted format, with the annotation information covering target categories and normalized bounding box coordinates. The vehicle detection dataset is divided into 3827 images for the training set, 1094 images for the validation set, and 547 images for the test set, according to the ratio of 7:2:1. The UA-DETRAC public dataset involving nighttime surveillance camera shots is chosen as the supplementary test data to validate the model’s performance in the vehicle type recognition task. The images in the UA-DETRAC dataset that are eligible for low illumination are filtered to construct a high-quality test dataset. The VDD-Light dataset effectively supports the model’s performance evaluation in real traffic surveillance scenarios and low-light environments.

The labeled vehicles are intercepted from the image to construct the low-light vehicle classification dataset VC-Data. Samples of the car class are downsampled, and data enhancement, including affine transformation, level flipping, Gaussian blurring, contrast adjustment, and brightness adjustment, is performed on the bus and truck class samples. The VC-Data dataset is divided into a training set and a test set in a ratio of 8:2, which serves as the basis for model training and validation. Table 2 sets out the vehicle classes and the corresponding number of images for the VC-Data dataset.

The self-built vehicle classification dataset VC-Data lays the foundation for evaluating the proposed low-light vehicle type recognition model’s performance. The self-built vehicle detection dataset VDD-Light supports the testing of the model’s performance.

## 5. Experiment

### 5.1. Implementation Details

Table 3 lists detailed experimental environments for this experiment. To fully utilize the computational resources and achieve the optimal training effect, the parameters of the TFF-Net model are set as follows: the Adam optimizer is used, the batch size is 1, the epoch is 100, the learning rate is set to 0.001 and reduced to 0.9 times the original one after 10 iterations, and the L2 regularization coefficient is 0.0001. The parameters for the YOLO are set as follows: using the SGD optimizer with a batch size of 64, an epoch of 100, and an initial learning rate set to 0.01. For the other comparison methods, the corresponding parameter adjustment and optimization are carried out according to their respective characteristics to ensure the best performance in the experiment.

### 5.2. Experimental Evaluation Metrics

Six metrics evaluate the model’s multi-classification and target detection task performance. Accuracy measures the model’s overall predictive accuracy. Precision (P) values the proportion of True Positive results in a prediction. Recall (Recall, R) quantifies the coverage of actual positive results. Mean Average Precision (mAP) assesses the robustness of the multi-class detection. The mAPs used include an average precision mAP50 at 50% of the intersection and union ratio (IoU) threshold and an average precision mAP50-95 in the range of 50% to 95% of the IoU threshold. The number of parameters (Params) indicates the spatial complexity of the model.

The calculation method of each evaluation index is shown in Equations (15)–(20).

(15) $$Accuracy=\frac{TP+TN}{TP+TN+FP+FN}$$

(16) $$P=\frac{TP}{TP+FP}$$

(17) $$R=\frac{TP}{TP+FN}$$

(18) $$F1=2\cdot \frac{P\cdot R}{P+R}$$

(19) $$AP=\int_{0}^{1}P(R)dR$$

(20) $$mAP=\frac{1}{C}\sum_{c=1}^{C}AP_{c}$$

where TP is the True Positive class and represents the number of classes that the model correctly predicts as positive; FN is the False Negative class and indicates the number of positive classes that the model incorrectly predicts as negative; FP is the False Positive class, which shows the number of negative classes that the model incorrectly predicts as positive; TN is the True Negative class and indicates the number of classes that the model correctly predicts as negative. AP denotes the area enclosed by the PR curve and the axes; C means the number of tested sample categories.

### 5.3. Results and Discussion

#### 5.3.1. Module Validity Experiments

Experiments are designed on the VC-Data dataset to validate the effectiveness of each module in the proposed model’s feature extraction and mining phase. The M1, M2, M3, M4, and M5 models are specified by sequentially removing the multi-scale convolutional kernel, the channel attention mechanism (ECA Block), the GCN convolutional layer (i.e., entirely using GAT layers), and the GAT convolutional layer (i.e., entirely using GCN layers). The accuracy is used as a criterion to verify the validity of the module sequentially, and the results are presented in Table 4.

Based on the experimental results, each module in the proposed model’s feature extraction and mining phase plays a vital role in the performance’s improvement. The multi-scale convolutional kernel improves the accuracy by 2.00% by capturing features at different scales, which proves the significance of an enhanced feature richness. Model accuracy decreases by 2.97% after the ECA channel attention mechanism is removed, highlighting its ability to screen key feature channels. Both GCN and GAT layers are used in conjunction with each other to improve the accuracy by 1.50% and 1.00%, respectively, compared to a single structure, which reflects the effectiveness of mining graph structure features with a mixture of GCNs and GATs. These modules complement each other in feature extraction, feature screening, information dissemination, and relationship mining, resulting in a 93.25% accuracy in recognizing vehicle types in low-illumination environments.

#### 5.3.2. Experiments on Overall Model Performance

**Figure 10:**
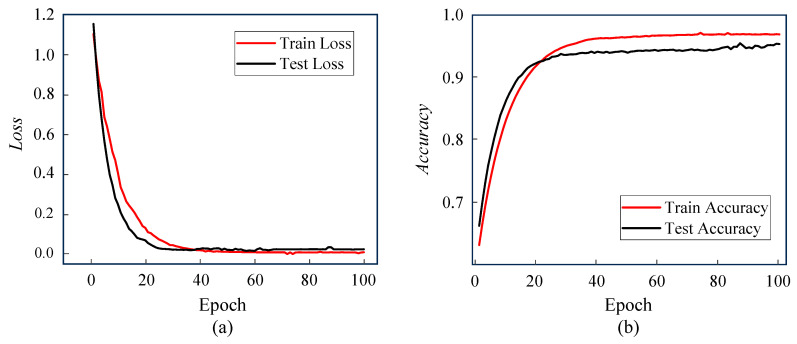
Loss and accuracy curves. (**a**) loss curve; (**b**) accuracy curve.

The overall vehicle type recognition model’s performance in low-illumination environments is trained and tested. The obtained loss and accuracy curves are presented in Figure 10.

From Figure 10a’s loss curves, Train Loss and Test Loss plummet and stabilize at the beginning of training (first 40 rounds). This shows that the model converges efficiently and without overfitting. Figure 10b displays that the training accuracy rises from 60% to nearly 90%, and the test accuracy increases simultaneously. The growth rate slows down and eventually stabilizes around 95% after 20 rounds, which reflects the model’s good generalizability. Together, both types of curves validate the data quality and model design.

After the experimental training and testing, the specific values of the evaluation indicators corresponding to each category (car, bus, truck) are obtained and summarized in Table 5.

**Figure 11:**
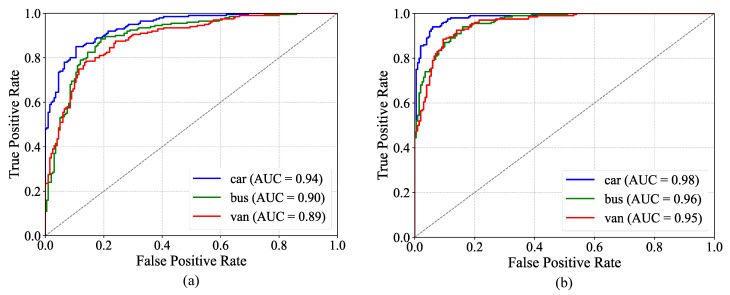
ROC curves. (**a**) ROC curve for the M5 model; (**b**) ROC curves for the TFF-Net model.

The ROC curves for the M5 model and the TFF-Net model are illustrated in Figure 11. Intuitively, from the curve morphology, TFF-Net makes a significant improvement in the classification performance of each category. The curves are more closely aligned towards the upper left corner, which means TFF-Net is able to recognize the positive case samples more accurately and reduce the occurrence of misclassification situations.

The low-light vehicle recognition model TFF-Net proposed in this paper shows excellent performance in the test set, with an overall accuracy of 0.9573. TFF-Net has optimal precision (0.9842), recall (0.9564), and F1 values (0.9701) in the car category. The F1 values for the bus and truck categories are 0.9467 and 0.9424, respectively, significantly narrowing the inter-category performance differences compared to the M5 model. It is shown by the multi-dimensional evaluation that the effectiveness and robustness of the proposed model for vehicle type recognition in low-light environments are fully verified.

#### 5.3.3. Ablation Experiments

Ablation experiments are performed on the VC-Data dataset to validate the effectiveness of each module in the overall TFF-Net model. The specific operation is to add feature fusion, AWF-Bagging, and the improved loss function sequentially based on the M5 model to constitute the M6, M7, M8, and TFF-Net models. Each module is verified in turn for its role in the network in terms of accuracy, and the results of the ablation experiments are shown in Table 6.

The experimental results show that the progressive optimization strategy in this paper significantly enhances the performance of the low-light vehicle recognition model. With the introduction of the multi-scale feature fusion module, the accuracy is improved by 1.08%, and the synergistic modeling of local features and global representations enhances the feature expression capability. The AWF-Bagging algorithm is further adopted to improve the accuracy by 0.84% to 95.17%. This algorithm effectively coordinates the performance differences among base classifiers through a dynamic weight allocation, strengthening the model’s ability to adapt to data heterogeneity. After the improved loss function is employed, the model accuracy reaches a peak of 95.73%, which suppresses overfitting while improving the robustness of classification in uncertain environments. The proposed model breaks through the limitations of traditional methods from the three dimensions of feature characterization, model generalization, and decision boundaries, respectively, and provides a systematic solution for vehicle recognition under complex lighting conditions.

#### 5.3.4. Comparison Experiments

Comparison experiments are designed to verify the effectiveness of the proposed vehicle type recognition model for low-light environments. The model’s performance is quantitatively compared to benchmark methods such as ResNet50, AlexNet, SVM-HOG, and Faster R-CNN. Table 7 shows the results of the model-specific comparisons.

The experimental results show that TFF-Net is significantly superior to the comparison method in all evaluation indexes. In terms of macro evaluation indicators, Macro-Precision, Macro-Recall, and Macro-F1 reached 0.9490, 0.9576, and 0.9531, respectively, which are all significantly improved. The results confirm the effectiveness of TFF-Net in dealing with the category imbalance problem and the model’s reliability in vehicle type recognition in low-light environments.

#### 5.3.5. Significance Analysis Experiments

**Figure 12:**
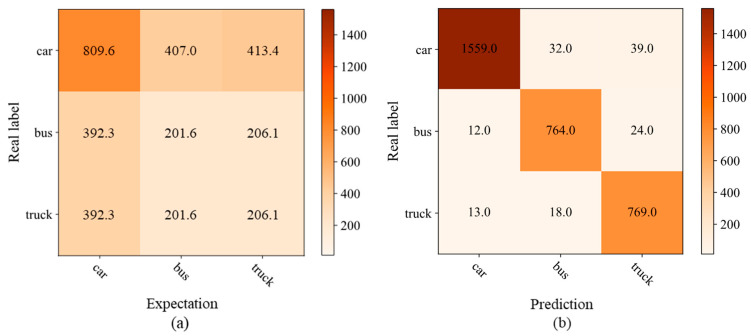
Expectation and prediction (confusion matrix). (**a**) expectation matrix; (**b**) prediction matrix.

A confusion matrix is a tool used to represent the classification results of the model on the test set. It can help to evaluate the performance of the model and analyze the type of errors. After experimental training and testing, the confusion matrix corresponding to each category (car, bus, truck) is obtained, as illustrated in Figure 12b. The expected frequencies under the assumption of random guessing are calculated, as shown in Figure 12a.

Calculating the chi-square statistic ($\chi^{2}$) based on the observed values (values in the confusion matrix) and the expected values yields $\chi^{2}=$ 5586.7 for a three-class classification problem with a degree of freedom of four, which ultimately computes a *p*-value much less than 0.05. Since the *p*-value is much less than 0.05, we reject the original hypothesis and conclude that there is a significant difference between the model’s classification results and random guessing. This indicates that the proposed model’s classification performance is significantly better than random guessing.

#### 5.3.6. Target Detection Performance Test Experiments

One-stage deep learning methods include the YOLO series [47,48], of which YOLOv11n [49] is the latest lightweight model. The original convolutional classification head of YOLOv11n adopts the regular mesh feature extraction mechanism, which suffers from an insufficient capability to model the topology among vehicle parts and the poor robustness of the features in low-illumination and occlusion scenes. To this end, this paper presents the target detection algorithm YOLOv11-TFF by introducing the proposed TFF-Net instead of the original classification head. YOLOv11-TFF follows the improved cross-entropy loss function. The algorithm combines the efficient localization capability of YOLOv11n with the graph neural network feature modeling advantage of TFF-Net to achieve the accurate positioning and reliable classification of vehicles in low-illumination environments.

The improved YOLOv11n algorithm is compared with the current mainstream target detection algorithms to verify the reliability and good detection performance of YOLOv11-TFF. Table 8 shows the experimental results of the comparison of Precision, Recall, mAP50, and the number of parameters (Params) for each algorithm on the test set.

Table 8 illustrates that YOLOv11-TFF performs outstandingly compared with other models, with a Precision of 0.958, Recall of 0.969, mAP50 as high as 0.987, and mAP50-95 of 0.914. This indicates that the model has less misclassifications and an accurate recognition and can effectively detect real vehicle targets. The proposed model has a high detection accuracy under different IoU thresholds, demonstrating adaptability and robustness. The number of parameters of YOLOv11-TFF is 4.26 × 106, which ensures high accuracy with reasonable complexity and achieves a favorable balance between accuracy and resource requirements.

**Figure 13:**
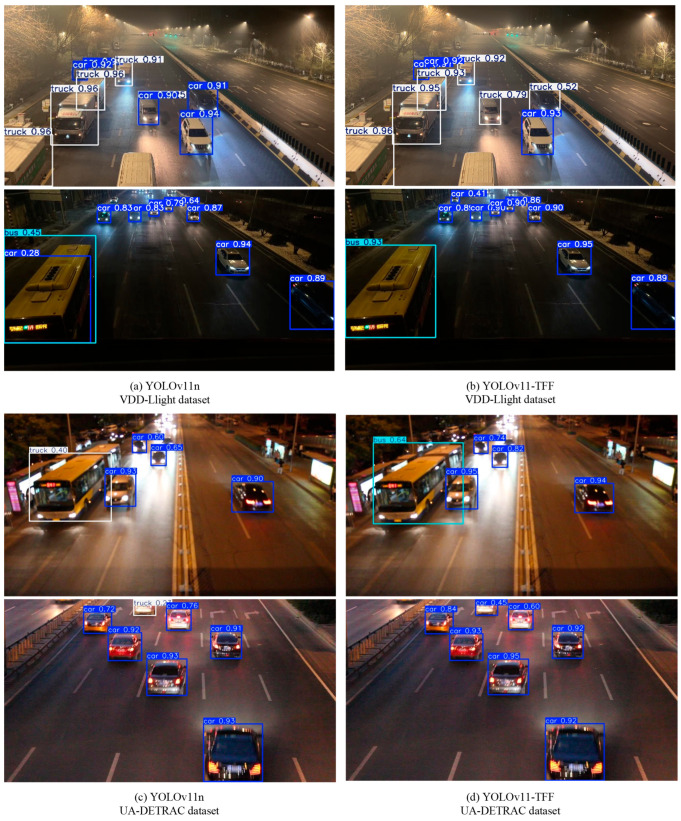
Comparison of target detection visualization results.

The performance of the YOLOv11-TFF algorithm is validated using the self-built VDD-Light low-light environment vehicle detection dataset and the UA-DETRAC public dataset. Figure 13 sets forth a comparison of the target detection results for YOLOv11n and YOLOv11-TFF.

As shown in the results of the visualization evaluation, YOLOv11-TFF is able to recognize models stably in a variety of low-light real-world application scenarios. The proposed method can correctly identify the vehicle type even in poorly lit areas or when the target vehicle is far away. It shows that YOLOv11-TFF is highly adaptable and stable in low-light environments, which lays a solid foundation for the algorithm’s large-scale promotion in practical applications.

## 6. Conclusions

This paper designs and optimizes a model using graph neural networks to address the shortcomings of existing vehicle type recognition in low-light environments. We build local–global two-stream feature extraction networks containing multilevel convolution and map the output features to nodes constructed as graph structures. A hybrid GNN architecture is designed to enhance feature expression. The output features of the GNN layer are fused to achieve vehicle type recognition and classification after the attention pooling module. Enhanced model stability and generalization are achieved using the improved adaptive weighting AWF-Bagging algorithm. Meanwhile, the dynamic adjustment weight mechanism and label smoothing technique are introduced to optimize the loss function and solve the category imbalance and overfitting problems. For the recognition and classification tasks, the TFF-Net model achieves an overall accuracy of 95.4% on the test set. After integration with the target detection algorithm, the YOLOv11-TFF algorithm achieves a mAP of 91.4%, with a model parameter count of 4.26 M. The proposed model can realize high-precision vehicle recognition and is expected to be deployed in edge computing devices, which verifies the practical value of the model in real traffic monitoring scenarios based on camera sensors. This paper also has some limitations, and the subsequent research will focus on making the model lightweight and applications in complex weather scenarios such as rain, snow, and fog.

## Figures and Tables

**Table 1:** Comparison of publicly available vehicle datasets.

Dataset	Year of Publication	Number of Images	Angle of the Shot		Collection Time
			Vehicle Camera	Monitor View	
KITTI [43]	2012	14,999	√	×	Day
Apollo Scape [44]	2019	140,000	√	×	Day/Night
BDD100K [45]	2018	100,000	√	×	Day/Night
UA-DETRAC [46]	2020	140,000	×	√	Day/Night

**Table 2:** Detailed information on the VC-Data dataset.

Type of Vehicle	Total Number of Samples	Number of Samples in Each Category	Number of Training Sets	Number of Test Sets
car	16,148	8148	6518	1630
bus		4000	3200	800
truck		4000	3200	800

**Table 3:** Experimental environment configuration.

Name	Specific Parameters	Company Name and Address
CPU	Intel(R) Xeon(R) Platinum 8474C	Intel Corporation, Santa Clara, CA, USA
GPU	NVIDA GeForce RTX 4090D	NVIDIA Corporation, Santa Clara, CA, USA
Memory	24 GB	NVIDIA Corporation, Santa Clara, CA, USA
Operating system	Windows10	Microsoft Corporation, Redmond, WA, USA
CUDA	11.8	NVIDIA Corporation, Santa Clara, CA, USA
IDE	Pycharm2021.3	JetBrains, Prague 9, Czech Republic
Programming languages	Python3.8	Python Software Foundation, Wilmington, DE, USA
Deep learning framework	Pytorch, PyG (PyTorch Geometric)	Meta Platforms, Inc., Menlo Park, CA, USA

**Table 4:** Module validity experiment results.

Model	Accuracy	Multi-Scale Convolution	ECA Channel Attention Mechanism	GCN Convolutional Layer	GAT Convolutional Layer
M1	0.9125	-	√	√	√
M2	0.9046	√	-	√	√
M3	0.9175	√	√	-	√
M4	0.9225	√	√	√	-
M5	0.9325	√	√	√	√

**Table 5:** Evaluation results of the TFF-Net model.

Type of Vehicle	Accuracy	Precision	Recall	F1
car	0.9573	0.9842	0.9564	0.9701
bus		0.9386	0.9550	0.9467
truck		0.9243	0.9613	0.9424

**Table 6:** Ablation experiment results.

Model	Accuracy	Feature Fusion	AWF-Bagging	Improved Loss Function
M6	0.9325	-	-	-
M7	0.9433	√	-	-
M8	0.9517	√	√	-
TFF-Net	0.9573	√	√	√

**Table 7:** Results of model comparison experiments. The proposed model and its experimental results are bolded.

Model	Accuracy	Precision			Recall			F1		
		Macro	Micro	Weight	Macro	Micro	Weight	Macro	Micro	Weight
ResNet50	0.9015	0.8883	0.8911	0.8937	0.8701	0.8732	0.8751	0.8791	0.8843	0.8821
AlexNet	0.8903	0.8748	0.8853	0.8806	0.8532	0.8603	0.8567	0.8644	0.8723	0.8669
SVM + HOG	0.8721	0.8502	0.8598	0.8553	0.8302	0.8364	0.8331	0.8403	0.8481	0.8442
Faster R-CNN	0.9250	0.9202	0.9355	0.9231	0.8952	0.9001	0.8983	0.9031	0.9084	0.9062
**TFF-Net**	**0.9573**	**0.9490**	**0.9573**	**0.9591**	**0.9576**	**0.9573**	**0.9573**	**0.9531**	**0.9573**	**0.9570**

**Table 8:** Results of comparative experiments on target detection models. The proposed model and its experimental results are bolded.

Model	Precision	Recall	mAP50	mAP50-95	Params/10^6^
YOLOv5s	0.919	0.964	0.969	0.854	2.19
YOLOv7n	0.923	0.932	0.947	0.907	6.02
YOLOv8n	0.931	0.937	0.956	0.917	2.70
Faster R-CNN	0.899	0.912	0.931	0.884	41.3
SSD	0.865	0.878	0.901	0.832	26.3
YOLOv11n	0.932	0.943	0.961	0.892	2.58
**YOLOv11-TFF**	**0.958**	**0.969**	**0.987**	**0.914**	**4.26**

## Data Availability

The original contributions presented in this study are included in the article. Further inquiries can be directed to the corresponding author.
